# Investigation of the Photocatalytic Activity and Light-Absorbing Properties of SrTiO_3_/TiO_2_NT@S Composite

**DOI:** 10.3390/molecules30234626

**Published:** 2025-12-02

**Authors:** Yelmira Nurlan, Aruzhan Chekiyeva, Arman Umirzakov, Madina Bissenova, Yerlan Yerubayev, Konstantine Mit

**Affiliations:** 1 Department of Materials Science, Nanotechnology and Engineering Physics, Satbayev University, Almaty 050013, Kazakhstan; 2 Institute of Physics and Technology, Almaty 050032, Kazakhstan; 3 Institute of Nuclear Physics, Almaty 050032, Kazakhstan; 4 Department of Food Production and Biotechnology, Taraz University Named After M.Kh. Dulaty, Taraz 080000, Kazakhstan

**Keywords:** TiO_2_ nanotubes, anodization, SrTiO_3_, S, photocatalysis, water treatment, degradation

## Abstract

This paper reports an assessment of the photocatalytic activity of TiO_2_ nanotubes (TNTs) doped with strontium titanate (SrTiO_3_) and sulfur (S) with respect to the decomposition of methylene blue (MB). TNT was obtained by the double anodizing method with further doping of strontium titanate by the hydrothermal method and additional annealing in an atmosphere of N_2_ (95%) + H_2_S (5%) at 450–550 °C. The photocatalytic activity was evaluated using MB as a pollutant and this study was conducted using an Osram Vita-Lux lamp with a power of 300W as a visible light source. The photocatalytic abilities of the synthesized materials were investigated, and characterized by methods such as SEM, TEM, XRD, EDS, and UV–Vis spectroscopy. Our study showed that the SrTiO_3_/TiO_2_NT@S composite has a better photocatalytic decomposition ability for the dye under consideration compared to pure TNT and SrTiO_3_/TiO_2_NT. These results clearly demonstrate the potential of synthesized SrTiO_3_/TiO_2_NT@S material for applications in water purification and photocatalysis.

## 1. Introduction

Titanium oxides have attracted considerable attention from researchers over the past two decades due to a number of their inherent physicochemical properties, such as high photocatalytic activity, chemical stability, non-toxicity, high resistance to corrosion and photocorrosion, durability and biocompatibility [1,2]. Currently, several types of TiO_2_ morphologies have been reported. However, nanostructured forms such as nanotubes, nanorods, and nanowires have received scientific attention due to the combination of the electrochemical and photochemical properties of the material with the structural parameters of nanoparticles [3,4,5]. These properties make them suitable for applications in areas such as photocatalysis, sensors, electrochemical devices, and biomedical implants [6,7,8,9]. Among the various methods of synthesis of titanium dioxide nanotubes, anodic oxidation is the most widely used and effective method that allows us to control the morphology of nanotubes by changing the anodizing parameters [10,11,12].

Despite these advantages, the wide band gap of TiO_2_ nanotubes (~3.2 eV) limits photoabsorption to the ultraviolet region of the spectrum, which reduces the efficiency of photoconversion and impedes their industrial applications [13,14]. Many studies have been aimed at improving the efficiency of photoconversion of TiO_2_ nanotubes. Among them are the coupling of TiO_2_ nanotubes with SnO_2_, CdS, and CdSe semiconductors [15,16,17,18,19], doping with light elements such as C or N [20,21], and the formation of ternary oxides (ABO_3_), such as perovskites, on the surface of TiO_2_ nanotubes [22,23]. Among various perovskite structures, strontium titanate (SrTiO_3_) is a promising material for photoelectrochemical and energy applications, which improves the transport of charge carriers and the stability of TiO_2_-based systems. The TiO_2_/SrTiO_3_ system works well in photocatalysis because it efficiently separates charge carriers at the surface where the two materials meet [24,25].

Strontium titanate is a cubic perovskite oxide with outstanding electronic and optical properties [26,27]. SrTiO_3_ is an n-type semiconductor that is widely used for the degradation of the organic pollutants, as well as for the photoelectrochemical water splitting [28,29]. However, SrTiO_3_ has a wide band gap (3.0–3.2 eV) just like a TiO_2_, which limits the ability to absorb ultraviolet radiation. This means that more solar radiation remains unused and the overall efficiency of photocatalysis decreases.

In order to increase photosensitivity and expand the spectral response, various modification methods are used, including doping with non-metals [30]. It has been reported that the addition of sulfur improves photocatalytic activity in the visible light region, since additionally formed intermediate levels under the conduction band provide more efficient charge separation [31]. There are various mechanisms of sulfur incorporation, such as substitution of titanium ions (cationic), which is energetically more favorable, or oxygen (anionic) [32,33]. Nevertheless, only a limited number of studies have investigated the effect of sulfur doping in composites based on SrTiO_3_ and TiO_2_.

**Figure 1:**
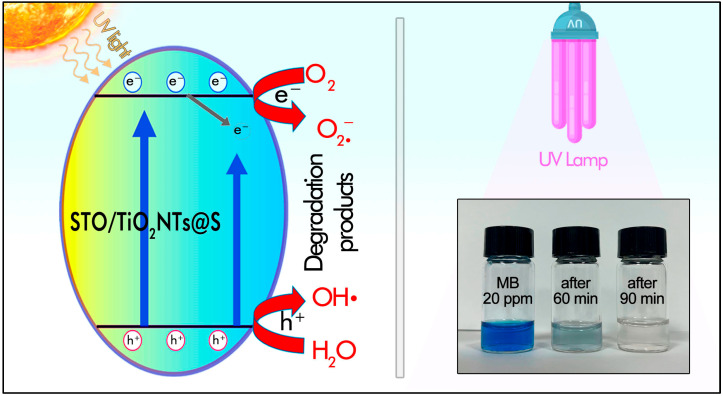
Photocatalytic mechanism and degradation of the model dye (methylene blue, MB) over SrTiO_3_/TiO_2_NT@S under UV light irradiation.

In this study, we present a simple and cost-effective method for synthesizing a composite that combines the advanced nanotube morphology of TiO_2_ with improved light-absorbing properties provided by SrTiO_3_ and S doping. The resulting photocatalytic material was characterized by analytical methods such as X-ray diffraction (XRD), transmission electron microscopy (TEM), energy dispersive X–ray spectroscopy (EDX), and UV-Vis spectroscopy. Figure 1 shows the mechanism of degradation of methylene blue under ultraviolet and visible irradiation, as well as the degradation and charge separation in the SrTiO_3_/TiO_2_NT@S composite.

## 2. Results and Discussion

**Figure 2:**
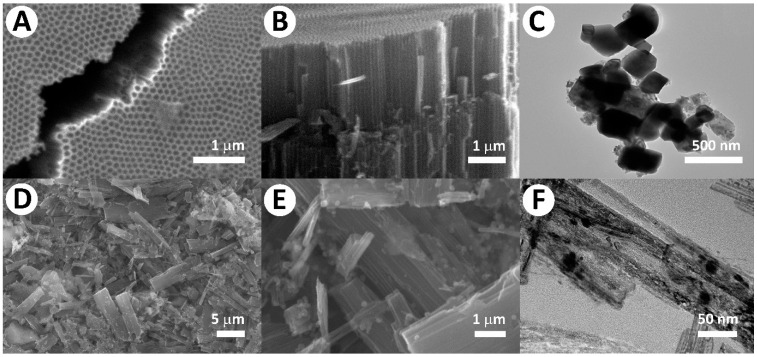
SEM morphology and cross-section images of: (**A**,**B**) obtained TNT; TEM images of: (**C**) obtained SrTiO_3_ [34]; (**D**,**E**) SEM images of: SrTiO_3_/TiO_2_NT; (**F**) TEM images of: SrTiO_3_/TiO_2_NT@S.

The morphology of the obtained nanotube structures shown in the SEM images of the samples after double anodization (Figure 2A,B), a highly ordered array of vertically oriented TiO_2_ nanotubes with a uniform diameter of ~80–120 nm and dense packing is formed. Such a hierarchical organization, characteristic of anodized arrays, provides a large specific surface area and direct pathways for charge transport, which is critically important for photocatalytic processes. Thus, the resulting nanotube arrays provide motivation for further functionalization and bonding (SrTiO_3_, S). Tight surface contact facilitates efficient carrier migration between the two semiconductors, suppresses recombination, and thus improves photocatalytic properties.

The TEM images (Figure 2C) clearly show well-formed SrTiO_3_ cubes, previously synthesized using the method described in a previous study [34]. The particles are anisotropic in nature and have an average size of ~200 nm, indicating high crystallinity and uniform growth. Following the hydrothermal treatment, TiO_2_ nanotube arrays and SrTiO_3_ particles (Figure 2D,E, respectively) maintain their structural integrity while forming close interfacial contact. An important feature of these particles is their anisotropic structure, which creates energy differences across the crystal facets, leading to the formation of p-n junctions which allows the charge inside each photocatalyst particle to be separated through the interfacial electric field.

In the SrTiO_3_/TiO_2_NT@S composite (Figure 2F), nanoparticles are uniformly distributed across the tube vertices and the intertubular space, forming multiple TNT/SrTiO_3_ interfacial contacts. This architecture facilitates efficient interfacial charge transfer (type-II or S-scheme scenarios are possible, depending on the composition and defective structure), reduces charge recombination, and thereby accelerates photodegradation of organic dyes. To extend the photosensitivity of the system to the visible region, either doping (for example, S-doped TiO_2_) or coupling with components that actively absorb in the visible range is commonly employed. According to literature data (XPS), during heat treatment above ~500–550 °C, the concentration of S- and N-dopants in TiO_2_ decreases markedly, while correctly performed S-alloying can be maintained at 400–500 °C. This aspect should be considered when choosing the temperature regime for processing composites.

**Figure 3:**
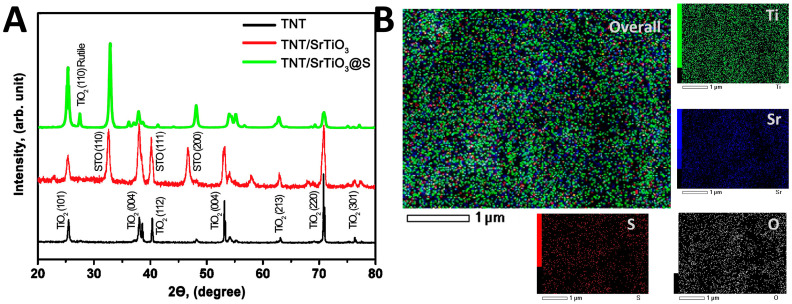
(**A**) XRD patterns of the referential and sulfur-modified TiO_2_-based photocatalysts and (**B**) EDX corresponding elemental mapping for Ti, O, Sr and S.

This well-organized morphology indicates successful anodization and provides a robust base for further structural and phase analysis. X-ray diffraction analysis of SrTiO_3_/TiO_2_NT@S samples was carried out on a diffractometer with scanning angles ranging from 20° to 80° and a step size of 0.01, as shown in Figure 3A. The characteristic diffraction peaks of TNT samples were indexed to the (101), (004), (112), and (220) planes, observed at 2θ = 25.4°, 37.9°, 39° and 70.3°, which indicates the polycrystalline structure of anatase, in good agreement with the standard map for TNT (JCPDS No. 21-1272) [35]. Additionally, X-ray diffraction revealed the appearance of new peaks at 32.2°, 36.9°, 47.8°, and were indexed to the (110), (111), and (200) planes, which relate to the cubic phase of SrTiO_3_ perovskite (JCPDS No. 35-0734). This confirmation is based on a comparison of the diffraction spectra of the composite with TNT, making it possible to determine whether changes have occurred in the crystal structure during their combination.

The crystal lattice parameters of the TiO_2_ and SrTiO_3_ phases were also calculated. The lattice constant of the cubic phase of SrTiO_3_ slightly increased from 3.803 Å to 3.825 Å after sulfur modification (Δa < 1%). This slight expansion of the lattice is associated with the formation of defects and sulfur-induced relaxation of internal stresses. The structural stability of the TiO_2_ anatase phase was confirmed by the fact that the crystal lattice parameters showed no significant changes.

It is especially important to note that the peak at 2θ = 71.23° has a high intensity, indicating a high degree of crystallinity in the semiconductor. This is important because the efficiency of charge carrier transfer formed during photogeneration can strongly depend on the crystallinity of the material. Low crystallinity can lead to inefficient migration of charged particles. The average crystallite size (D) was calculated using the Scherrer Equation (1):

(1) $$D=\frac{k\;\lambda }{\beta cos\;\theta },$$

where *k* is a constant (=0.9), λ is the Cu Kα radiation wavelength (0.154 nm), β is the line width (in radians), and θ is the angle of diffraction. The calculated crystallite sizes were about 18 nm for TiO_2_ (anatase), 20 nm for SrTiO_3_, and 22 nm for the S-doped SrTiO_3_/TiO_2_NT@S composite. All calculations were performed for the most intense reflections of each phase -(101) for TiO_2_ anatase and (110) for cubic SrTiO_3_. A slight increase in crystallite size can be observed after the introduction of SrTiO_3_ and sulfur doping, which is probably due to interfacial stress and defect-induced lattice distortions leading to partial relaxation of the structure during heat treatment. We also observe a shift in the diffraction peak compared to the unmodified TNT/SrTiO_3_ composite after modification with sulfur. This shift toward lower 2θ values indicates an increase in the interplanar spacing and, therefore, a change in the parameters of the SrTiO_3_ crystal lattice. This kind of behavior may result from the incorporation of sulfur atoms into the anionic sublattice of SrTiO_3_, which partially replaces oxygen, or the formation of defects such as oxygen vacancies, as well as internal stresses occurring at the SrTiO_3_/TiO_2_ interface. Together, these factors lead to a slight expansion of the SrTiO_3_ lattice, which is reflected in the observed peak shift [36,37].

The structural and morphological observations were further supported by elemental mapping analysis (EDS). The EDX elemental mapping (Figure 3B) revealed a uniform distribution of Ti and O, as well as the dispersed presence of Sr and S without agglomeration. Quantitative analysis confirmed the atomic ratios of Ti (49.56%) and Sr (50.44%), indicating the successful formation of a composite with multiple interfacial contacts. The unusual stoichiometry of the composite is explained by the local surface composition rather than the bulk atomic ratio. The signals from SrTiO_3_ particles primarily originate from TiO_3_ deposited on the nanotube walls, while Ti is present from both the TiO_3_ substrate and the SrTiO_3_ phase. Consequently, we observe a similar Ti:Sr ratio, indicating extensive coverage of the SrTiO_3_ nanotube surface.

Additional sulfur doping (SrTiO_3_/TiO_2_NT@S) leads to a change in the diffraction pattern: there is a broadening of individual TiO_2_ lines and intensification of SrTiO_3_ signals. This is due to the incorporation of sulfur atoms into the TiO_2_ lattice and the formation of defective states that cause local distortions of the crystal structure. Such modifications alter the electronic structure and the expansion of the absorption spectrum into the visible light region. Thus, the XRD results confirm the successful formation of the ternary SrTiO_3_/TiO_2_NT@S composite, which combines high crystallinity, stable heterointerfaces, and structural distortions caused by S-doping. These factors create prerequisites for improved photoelectronic characteristics and increased photocatalytic activity of the material.

**Figure 4:**
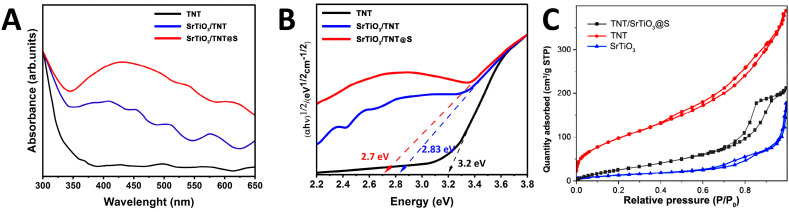
(**A**) UV−vis absorbance spectra (**B**) and Tauc plots of TNT, TNT/SrTiO_3_ and SrTiO_3_/TiO_2_NT@S samples (**C**) Nitrogen adsorption–desorption isotherms of TNT, SrTiO_3_, and SrTiO_3_/TiO_2_NT@S samples measured at 77 K.

To clarify the influence of SrTiO_3_ coupling and sulfur doping on the optical response, UV–Vis diffuse reflectance spectra of unadulterated TiO_2_ nanotubes, SrTiO_3_/TiO_2_NT, and SrTiO_3_/TiO_2_NT@S composites were obtained. Figure 4A shows the diffuse reflection spectra (UV–Vis) for TNT, TNT/SrTiO_3_, and SrTiO_3_/TiO_2_NT@S samples. Pure TNT exhibits absorption mainly in the ultraviolet region (300–370 nm), which is consistent with the wide band gap of anatase (~3.2 eV). After the modification of TNT with SrTiO_3_ nanoparticles (blue curve), the spectrum is expanded to the region of 350–500 nm, which is associated with the formation of a composite and more efficient interfacial transfer of charge carriers. The SrTiO_3_/TiO_2_NT@S composition demonstrates the most pronounced absorption in the visible region (400–650 nm). Sulfur doping modifies the TiO_2_ electronic structure by introducing impurity levels, thereby further increasing the absorption of visible light [38]. The band gap of the samples could be estimated from the absorption edges. The band gaps, estimated from the absorption edges, were ~3.23 eV (TNT), ~2.85 eV (TNT/SrTiO_3_) and ~2.70 eV (SrTiO_3_/TiO_2_NT@S). These results indicate that the combination of SrTiO_3_ and sulfur enhances light absorption and increases the sensitivity of the material to visible radiation.

The decrease in the optical band gap is due to the synergistic interaction of interface electron bonds, defect-related states, and local band bending at the interfaces. Due to dense interfaces, SrTiO_3_/TiO_2_ induces orbital hybridization between the Ti-3d and O-2p states, which leads to the appearance of intermediate electron levels near the conduction and valence bands. Simultaneously, the presence of sulfur impurities, oxygen vacancies, and Ti^3+^ centers leads to the appearance of localized intraband states, which serve as additional excitation channels in visible light. The internal electric field created by the equilibrium Fermi levels and the lattice mismatch further modifies the band edges, facilitating optical transitions in the subband region and improving charge separation. Consequently, photons with lower energies can be absorbed, resulting in an effective band gap. This behavior is consistent with recent studies [39] using ultraviolet photoelectron spectroscopy (UPS) and XPS, which have shown that the apparent narrowing of the optical band gap in oxide heterostructures is primarily due to surface potential modulation and interface hybridization effects rather than bulk electronic rearrangement.

A comparative analysis of UV–Vis spectra and Tauc plots Figure 4B confirms TNT modification using SrTiO_3_ and sulfur has a synergistic effect: the absorption range in the visible light region expands, the band gap decreases, and the probability of effective generation and separation of charge carriers increases.

The increase in photocatalytic activity after sulfur doping is not very large, but it is in line with the observed optical red shift and lower recombination tendency. This suggests that adding sulfur affects surface electronic states and charge transfer at the interface. Consequently, the influence of sulfur must be regarded as a cumulative yet consistent enhancement that facilitates improved photoresponse in visible light.

N_2_ adsorption–desorption measurements (Figure 4C) exhibit type IV isotherms with H3-type hysteresis loops, characteristic of mesoporous materials with slit-like pores formed by stacked or aggregated nanostructures. The TNT sample exhibits the highest surface area, approximately 103 m^2^/g, which corresponds to its highly developed nanotubular morphology. Pure SrTiO_3_ exhibits a significantly lower value, around 24 m^2^/g, which is typical of cubic perovskite particles with a relatively compact morphology. The SrTiO_3_/TiO_2_NT@S composite exhibits an intermediate specific surface area in the range of 58 m^2^/g, reflecting the partial coating of TiO_2_ nanotubes with SrTiO_3_ nanocubes and sulfur-containing compounds. Although the SrTiO_3_/TiO_2_NT@S composite does not have the largest surface area, it exhibits the highest photocatalytic activity, confirming that the increase in reaction efficiency is primarily due to interfacial charge transfer effects and band structure modification rather than surface area-dependent factors.

**Figure 5:**
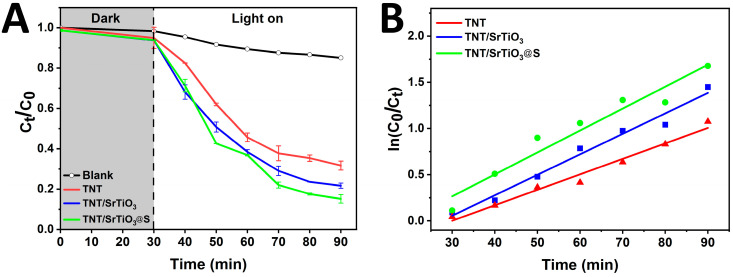
(**A**) Photocatalytic degradation and (**B**) kinetic curves of MB over TNT, TNT/SrTiO_3_, and SrTiO_3_/TiO_2_NT@S under visible light.

The photocatalytic activity of the TNT, TNT/SrTiO_3_, and SrTiO_3_/TiO_2_NT@S samples was evaluated by monitoring the temporal variation of the Ct/C_0_ ratio (Figure 5A), where C_0_ and Ct represent the initial and instantaneous concentrations of methylene blue, respectively. The kinetics of MB photodegradation are presented in Figure 5B. The rate of the photocatalytic reaction was considered to correspond to the pseudo–first-order reaction described by the Langmuir-Hinshelwood kinetics (2):

(2) $$ln\;(\frac{C_{0}}{C})=kt$$

where *C*_0_ and *C* are initial and final concentrations of MB solution in the moment of time—*t*, and *k* (min^−1^) is the rate constant of the reaction.

The calculated rate constants k = 0.028, 0.024, and 0.016 min^−1^ for SrTiO_3_/TiO_2_NT@S, TNT/SrTiO_3_, and TNT samples, respectively, presented in a Table 1 showed that the SrTiO_3_/TiO_2_NT@S composite has the highest photocatalytic activity. It is well known that TiO_2_ doped with S leads to a shift of the TiO_2_ absorption edge to the region of lower energies. This effect indicates that sulfur doping effectively suppresses the recombination of photogenerated electron-hole pairs [40] and enhances charge carrier transfer through the TiO_2_/SrTiO_3_ interface, leading to increased photocatalytic activity upon visible light irradiation. Researchers [41,42] have reported similar effects on electron–hole separation for TiO_2_/SrTiO_3_ composites. It has been shown that S-doped materials have slightly enhanced photocatalytic activity in the decomposition reactions of 2-propanol and MB when illuminated with visible light. Increased light absorption in the visible region is associated with the mixing of energy levels O 2p and S 3p [43,44]. Thus, the SrTiO_3_/TiO_2_NT@S composite can be considered as a promising photocatalyst for wastewater treatment under the influence of visible radiation. These results demonstrate the outstanding potential of SrTiO_3_/TiO_2_NT@S as a photocatalyst for the degradation of organic pollutants [45].

## 3. Materials and Methods

### 3.1. Materials

Ti foil (99.9%, thickness, 0.1 mm; China), ethylene glycol (99.9%, Kazakhstan), ammonium fluoride, strontium titanate powder synthesized using the procedure described in our previous work [35], hydrogen sulfide, ethanol, methylene blue.

### 3.2. Nanotube Synthesis

Titanium dioxide nanotubes were synthesized by a two-step anodization process. The titanium foil was used as the anode, and a platinum plate was used as the cathode. Before the anodization process, the samples were first cleaned by ultrasound in distilled water, after which chemical purification was carried out in a solution containing acetone, ethylene glycol, and distilled water. Anodizing was performed in an electrolyte consisting of 2% distilled water, 0.5% ammonium fluoride (NH_4_F), and ethylene glycol at a voltage of 60 V for 2 h at room temperature under constant magnetic stirring.

After completing the first stage of anodizing, the samples were washed in an ultrasonic bath in distilled water for 30 min. Then, the repeated anodization process was carried out under the same conditions. The obtained samples were dried at a temperature of 150° C for 2 h.

### 3.3. Synthesis of SrTiO_3_/TiO_2_NT@S

**Figure 6:**
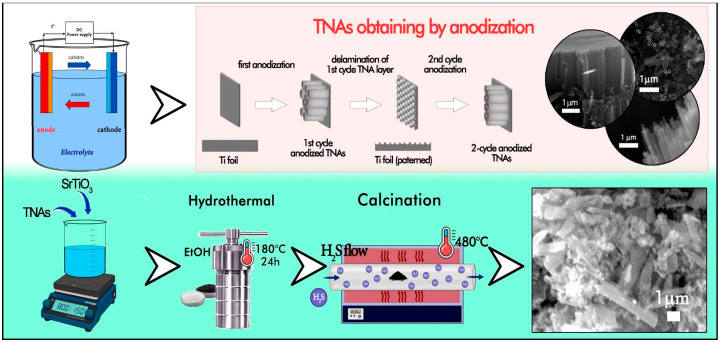
Schematic illustration of the stages of obtaining a composite.

TNT was used for the synthesis of the composite; strontium titanate powder obtained by the chemical method [45] was mixed in a mass ratio of 1:6. Ethanol was added to the resulting mixture, which served as a dispersion medium, after that mechanical mixing was carried out on a planetary ball mill using zirconia balls with a diameter of 1.0–1.2 mm; the use of small-diameter balls was due to the need to preserve the structure of the nanotubes. The mass ratio of powders and balls was 0.4:10 g. The mixture was stirred at a speed of 750 rpm for 3 h, resulting in a homogeneous white suspension. Next, the suspension was subjected to repeated centrifugation at 2500 rpm for 20 min at room temperature, then the precipitate was dried at 80 °C for 24 h and calcined at 480 °C for 15 min. After drying, the resulting white powder was subjected to additional annealing in an atmosphere of N_2_ (95%) + H_2_S (5%) at 450–550 °C for 10 min (heating rate of 10 °C/min), resulting in the formation of S-alloyed material; subsequent natural cooling for 3 h ensured the stabilization of its structure (Figure 6). We chose a temperature range of 450–550 °C to find a good balance between how well sulfur was added and how stable the structure was. Sulfur doping is not finished below 450 °C, and above 550 °C, TiO_2_ may lose its dopant and change from anatase to rutile. This time period makes sure that S-doping works while keeping the nanotubular shape and crystallinity.

### 3.4. Photocatalytic Measurements

The photocatalytic activity of SrTiO_3_/TiO_2_NT@S samples was studied by the decomposition of methylene blue under the influence of visible radiation from an Osram Vita-Lux lamp with a power of 300 W, simulating sunlight (the intensity on the surface of the solution was 15 mW/cm^2^). According to radiometric measurements, the lamp emits approximately 3.0 W in the UVB range (280–315 nm) and 13.6 W in the UVA range (315–400 nm), corresponding to a total UV radiation in the 280–400 nm range of about 16.6 W. Based on the measured total irradiance of the solution surface (15 mW cm^−2^), this corresponds to an estimated UV contribution of 0.83 mW cm^−2^, i.e., approximately 5–6% of the total incident radiation power in our experimental geometry at a lamp-to-sample distance of 7 cm. For the experiments, 29 mg of the composite was added to 20 mL of an aqueous solution of methylene blue (20 mg/l) and stirred for 30 min in the dark to establish an adsorption–desorption equilibrium. We found that a catalyst loading of 29 mg per 20 mL of solution (1.45 g·L^−1^) was a good balance between enough photocatalytic activity and not too much light scattering. After equilibration, the solution was irradiated, and every 30 min, 2 mL samples were taken, centrifuged (4000 rpm for 5 min) to get rid of catalyst particles, and then analyzed by UV–Vis spectrophotometry. Before the measurements, the suspension was centrifuged to remove solid particles of the catalyst, and the filtrate was examined using an SF-56 UV-visible spectrophotometer (LOMO, St. Petersburg, Russia). All tests were repeated to confirm the reproducibility of the results.

## 4. Conclusions

This paper proposes a simple synthesis method for a three-component composite, SrTiO_3_/TiO_2_NT@S, with photocatalytic properties. The materials were characterized by a set of techniques to determine their structure and composition. SEM and TEM identified arrays of vertically oriented TiO_2_ nanotubes (80–120 nm in diameter) decorated with cubic SrTiO_3_ nanoparticles (~200 nm) evenly distributed over the tube surfaces. Elemental analysis (EDX) confirmed a homogeneous distribution of Ti, O, Sr, and S without signs of aggregation, as well as dense interfacial contacts that promote efficient charge transfer. X-ray diffraction (XRD) revealed that the anatase phase of TiO_2_ was preserved, together with characteristic reflections of SrTiO_3_, indicating successful composite formation. UV–Vis spectroscopy and Tauc-plot analysis showed a decrease in the band gap from 3.23 eV (TNT) to 2.70 eV (SrTiO_3_/TiO_2_NT@S) and an extension of the absorption window to 400–650 nm. The samples were tested for methylene blue (MB) degradation to evaluate their photocatalytic performance. SrTiO_3_/TiO_2_NT@S exhibited a higher degradation rate than TNT/SrTiO_3_ and TNT specifically, the apparent rate constant under SrTiO_3_/TiO_2_NT@S was 1.75 times that of TNT.

## Figures and Tables

**Table 1:** The parameters for the pseudo-first-order photocatalytic reaction of TNT, SrTiO_3_/TiO_2_NT, and SrTiO_3_/TiO_2_NT@S.

The Sample	k, min^−1^	R^2^
TNT	0.016	0.96
SrTiO_3_/TiO_2_NT	0.024	0.98
SrTiO_3_/TiO_2_NT@S	0.028	0.98

## Data Availability

The data are contained within the article.
